# Contingency Management and Deliberative Decision-Making Processes

**DOI:** 10.3389/fpsyt.2015.00076

**Published:** 2015-06-01

**Authors:** Paul S. Regier, A. David Redish

**Affiliations:** ^1^Graduate Program in Neuroscience, University of Minnesota, Minneapolis, MN, USA; ^2^Department of Neuroscience, University of Minnesota, Minneapolis, MN, USA

**Keywords:** decision-making, deliberation, addiction, contingency management, neuroeconomics, impulsivity, addiction treatment

## Abstract

Contingency management is an effective treatment for drug addiction. The current explanation for its success is rooted in alternative reinforcement theory. We suggest that alternative reinforcement theory is inadequate to explain the success of contingency management and produce a model based on demand curves that show how little the monetary rewards offered in this treatment would affect drug use. Instead, we offer an explanation of its success based on the concept that it accesses deliberative decision-making processes. We suggest that contingency management is effective because it offers a concrete and immediate alternative to using drugs, which engages deliberative processes, improves the ability of those deliberative processes to attend to non-drug options, and offsets more automatic action-selection systems. This theory makes explicit predictions that can be tested, suggests which users will be most helped by contingency management, and suggests improvements in its implementation.

## 1 Contingency Management

Contingency management is a method of driving behavioral change through reinforcement with tangible rewards (1). It has been shown to significantly reduce drug-using behavior and increase continuous abstinence rates (2–9).

There are two main variations of contingency management, voucher-based and prize-based. In voucher-based treatment, patients are awarded points that accumulate for submission of drug-negative urine samples (3–5, 8). These points start out very low and can be exchanged for merchandise at any time. For example, in the Higgins et al. (5) study, points for the first clean sample were worth $2.50 and each subsequent sample was worth $1.50 more. By the end of the first month, a drug-negative sample was worth $16.50.

In prize-based treatment, patients earn a chance to win a prize with each drug-negative sample (1, 9–12). Typically, in these studies, prizes were worth around $1, $5, $20, and $100, and the probability to win higher-valued prizes was lower than lower-valued prizes (0.4% for a $100 prize and 68% for a $1 prize). Overall, the chance of the drug-negative sample having a monetary value of anything over a dollar was <7%.

## 2 Current Theories: Alternative Reinforcement

The success of contingency management is thought to be primarily due to the reinforcing properties of an alternative reward that is offered to patients for remaining abstinent (1, 5). The conceptualization of contingency management is that drug consumption is much like any other consumption of goods, and thus that increasing the cost of drugs should decrease use. Contingency management increases the cost of drugs because it creates an opportunity cost that is lost (the alternative reinforcer) when the user takes drugs. Reasoning for this is based on operant conditioning theories, noting that targeted behaviors increase with reinforcement and decrease in the presence of substitutes (13–16).

**Figure 1:**
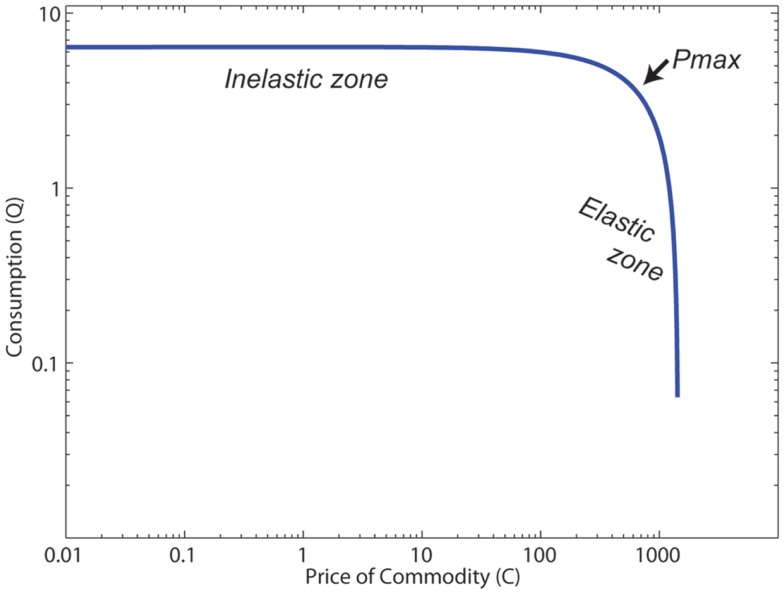
**The canonical structure of a demand curve**. *Pmax* is the point at which elasticity *E* = −1, and the elasticity transitions from inelastic (|*E*| < 1) to elastic (|*E*| > 1).

In economic terms, this change in use with cost can be measured as *elasticity*, which can be quantitatively defined as the change in the number of choices selected as cost increases (17–21). To determine this, one can measure the amount of effort an agent is willing to expend in order to gain the reward as a function of the cost. The function that results is called the *demand curve* (see Figure 1). A commodity that decreases quickly with cost is said to be “highly elastic,” while a commodity that decreases slowly with cost is said to be “inelastic.” (See Table 1 for definitions of the behavioral/neuroeconomic concepts used in this article.)

**Table 1:** **Economic theoretical constructs used in this article**.

**• Agent**: a decision-maker, whether it be human or non-human animal or a computer algorithm
**• Deliberation**: deciding between multiple options based on a search-and- evaluation process in which the two options are considered and compared. Deliberation depends fundamentally on the ability to imagine future outcomes
**• Demand curve**: a quantitative measure of elasticity, measuring the amount of an option selected as a function of the cost. Typical demand curves have an inelastic section, which transitions non-linearly to a highly elastic section as cost increases
**• Elasticity**: the idea that as costs increases, the selection of an economic object decreases. A thing that decreases quickly with cost is said to be “highly elastic,” while a thing that decreases slowly with cost is said to be “inelastic”
**• Opportunity cost**: alternative rewards lost by selecting a given option (selecting the given option removes the opportunity to select the alternative; the more valuable the alternative, the larger the opportunity cost)
**• Preference reversal**: a phenomenon in which the agent prefers one delayed choice over another delayed choice when they are both far in the future, but switches to prefer the second choice when that second choice becomes more immediate
**• Value**: the idea that a given option has an underlying utility for an agent. However, value has to be measured, for example, by a willingness-to-pay or by revealed preference
**• Willingness to pay**: a measure of the valuation of an object as a function of the amount of money or effort an agent is willing to put into achieving it
**• Revealed preference**: a measure of valuation of an object as a function of whether it is preferred or not when given in contrast to another option
Experiments find these measures can produce incompatible outcomes.

Quantitatively, the effectiveness of an alternative reinforcement depends on the elasticity of the drug that the alternative reinforcer is substituting for. Although early descriptions of drug use assumed that drugs were taken irrespective of cost, Becker and Murphy (17) pointed out that drugs were economic objects, and, as such, should show elasticity. While there are theoretical reasons to expect differences in the elasticity between drugs and natural rewards (17, 22), nevertheless, drugs do show elasticity both in non-human animals (23–27) and in humans (28–32). This means that increasing the cost (or increasing the size of the alternate options, which increases the opportunity cost) of taking the drug should decrease use. *Alternative reinforcement theory* predicts that the change in drug use from contingency management should be proportional to the elasticity of drug use.

As reviewed above, contingency management provides relatively low-value monetary rewards for abstinence (especially in the first month of treatment). For example, in voucher-based contingency management, rewards are as low as $2.50 for the very first negative urine sample and $16.25 for a negative sample after remaining abstinent the entire first month (5). The pre-clinical experiments suggest that the value of alternative reinforcement rewards used in contingency management should not reduce drug consumption as much as it does. The pre-clinical experiments suggest that either cost of the drug or magnitude of the reinforcer would need to be significantly higher than what is typically used in contingency management if alternative reinforcement alone were to account for the observed reductions of drug use in contingency management studies.

## 3 The Problem with the Alternative Reinforcement Theory

If we assume that drugs are economic objects, and thus are subject to change in demand or price, then one way to quantitatively measure level of consumption as a function of price is with a *demand curve*. The demand curve measures a fundamental concept of consumption: as price of the economic object increases, the consumption of that object will decrease (33, 34).

Figure 1 shows the structure of a typical demand curve. These curves can be well-fit with Eq. 1 measuring the relationship of the cost of some commodity (*C*) and the consumption of that commodity (*Q*) (35):

(1) $$Q=LC^{b}-e^{-\mathrm{aC}}$$

where *L* measures consumption at *C* = 1, and *b* and *a* are variables that relate to slope and acceleration of the slope, respectively. The slope of the curve predicts the elasticity of the commodity.

(2) E=b−aC

*Pmax* is the point at which the elasticity *E* = −1, which is the point at which elasticity transitions from <1 unit of decreased use per unit of increased cost (inelastic) to more than 1 unit of decreased use per unit of increased cost (highly elastic). Because the elasticity terms *a*, *b*, and the cost *C* appear in the exponents in Eq. 1, once the cost crosses *Pmax* [when *C* > (*b* + 1)/*a*)], consumption drops off very quickly. Using demand curves, we can construct a quantitative model to determine how monetary rewards should affect consumption of a drug. As mentioned previously, monetary values early in treatment are relatively low, and demand curve modeling suggests that these rewards alone would affect consumption of the drugs very little.

### 3.1 Modeling contingency management: The monetary value of vouchers early in contingency management treatment should have a negligible effect on the consumption of cocaine

Bruner and Johnson (21) constructed demand curves for individuals that regularly use cocaine by asking subjects how much cocaine they would buy as the cost increased. As noted above, providing alternative rewards increases the cost of the commodity (here the drug) through lost opportunities (an *opportunity cost*) – if the person takes the drug, then they do not get the alternative reward. This means that we can use these demand curves to predict how this opportunity cost should change the choices made.

Individuals in treatment get a voucher value of $2.50 the first time they provide a clean sample^1^. Using the assumption that individuals seeking treatment spend an average of $99/day (Petry, personal communication) during a typical day of cocaine use, and given that 1 unit of reward in the Bruner and Johnson (21) data was worth $5 on the street, a starting contingency management reward value of $2.50/day is worth approximately $0.13/unit.

A shift of $0.13/unit on the demand curve would be predicted to produce a negligible effect on cocaine consumption [the Bruner and Johnson (21) demand curve predicts a 1.6% change]. Even at the end of the first month of contingency management treatment, when patients receive a voucher worth $16.25 ($0.82/unit), there should be little change in consumption [the Bruner and Johnson (21) demand curve predicts a 17% change].

**Figure 2:**
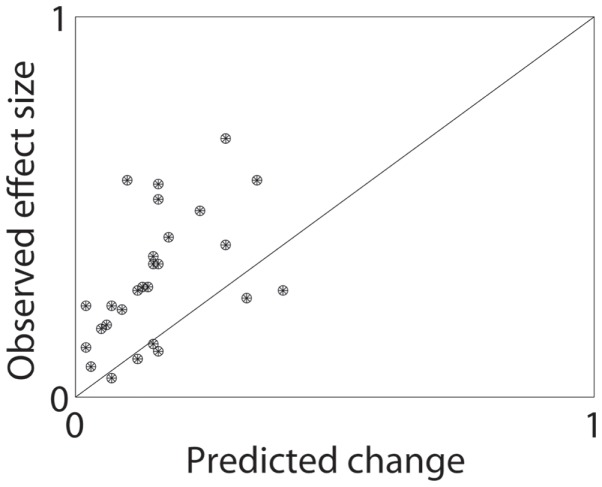
**Predicted and observed effect sizes of contingency management processes**. From the meta-analysis by Lussier et al. (36), we calculated the expected change in demand by applying the contingent alternate reward (in $) to the average demand curve found by Bruner and Johnson (21). This gave a predicted effect size, which was dramatically less than the typical effects observed. See text for additional discussion.

In order to quantitatively measure whether these economic changes could explain contingency management’s effects, we took the effect sizes reviewed in the meta-analysis by Lussier et al. (36) and asked how much the Bruner and Johnson (21) demand curve would predict from the economic change in cost alone. Of course, patients seeking treatment have increased costs for drug use due to many factors beyond the simple loss of the contingent alternate reward. Similarly, there is a large variability in how contingency management studies are run and what additional treatments they are paired with. Finally, the Bruner and Johnson (21) analysis is from one set of cocaine addicts, while the studies reviewed by Lussier et al. (36) range from alcoholics to stimulant addicts. Nevertheless, 21/27 studies had predicted changes less than the observed effect size, and the median ratio was that the predicted effect was less than half the observed (median ratio = 0.43). Figure 2 shows the distribution of observed effect sizes against the economically predicted changes. The predicted changes are significantly less than the observed changes (matched pairs median test, *p* = 0.00008).

This analysis suggests that the simple economic description of contingency management is inadequate – the rewards offered in contingency management are too small to have the observed effects. We suggest that this is because the microeconomic model on which the economic explanation for contingency management is based is inadequate – human decision-making depends on more than simple cost-benefit analyses. Instead, the human decision-making process is better described as an interaction between multiple competing components (37–43), each of which uses different processes to combine reward information (value) with past experiences (memory) to select actions (make decisions). We suggest that contingency management taps into certain aspects of these multiple decision-making systems to drive behavior to be more likely to reject the drug-taking choice.

## 4 Valuation

Early psychological and economic research postulated that reinforcers are *transituational*, meaning that the efficacy of the reinforcer remains consistent across different experimental conditions (44–46). However, studies have shown that reinforcers do not consistently elicit reliable behavioral outputs in different contexts (47).

In the fields of behavioral and neuroeconomics, decisions are assumed to derive from an underlying “value” or “utility” placed on outcomes. However, this value cannot be directly observed experimentally, and thus must be interpreted from experimental conditions. The two primary methods for deriving this value are *willingness to pay* experiments, in which an agent is given an opportunity to pay a cost for an outcome, and *revealed preference* experiments, in which an agent is given a choice between two or more options. In willingness-to-pay experiments, the agent has to decide whether to continue to pursue a given option or not. In revealed-preference experiments, the agent has to decide which option to pursue. Importantly, experiments in rats, monkeys, and humans all find differences between how animals value options under these two measurements, often finding incompatible outcomes (43, 47–49). Thus, converting experiments from single option (*Go or don’t?*) to multiple option (*Which one?*) can change how animals appear to value a given option.

A typical willingness-to-pay experiment would be the *breakpoint* procedure, in which an animal presses a lever to receive reward. The first reward is delivered with only a single-lever press, but the second requires two-lever presses, the third requires four, the fourth eight, and so on, doubling each time. At some point, the cost becomes too high and the animal stops pressing the lever (48, 50–52). In humans, willingness-to-pay can be assessed by simply asking “how much would you pay for this outcome?” (47).

By contrast, a typical revealed-preference experiment would provide an animal two levers, one of which provides one type of reward (A), while the other provides another type of reward (B) (48, 52, 53). The animal is only able to select one lever at any given time and thus must choose between the separate options. The implication is that the selected option is more valuable than the non-selected option. In humans, revealed preference can be assessed by asking “which option would you prefer?” (47).

Extensive evidence exists within the behavioral and neuroeconomics literature that these two measures can produce incompatible valuations, in which human subjects may be willing to pay more for option A than for option B, even when they would prefer to take option B when faced with the two options together (47). Recently, Ahmed (48) found in self-administering rats that measuring value by means of a breakpoint procedure (willingness-to-pay) can produce different ordering than when measuring value by means of a choice procedure (revealed preference); that is, subjects were willing to pay more for drug than saccharin but preferred saccharin to drug when given the choice (48). This strongly suggests that value is not an intrinsic (transituational) property, but is highly dependent on the contextual surrounding components.

These analyses implies that single-option experiments, in which an agent is tasked with deciding whether to pursue a given object or not may access different process than multiple-option experiments, in which an agent is tasked with deciding which option to pursue.

### 4.1 Valuation inconsistencies arise from multiple decision-making systems

Current theories suggest that this underlying lack of transsituationality arises because animals (including humans) make decisions based on several incompatible decision-making systems, each of which processes information about the decision in fundamentally different ways. Because these different systems drive behavior at different times, the same agent can show different valuations under different experimental conditions.

Classically, the idea that valuation is inconsistent and not transsituational has been addressed in terms of *dual-process theories* that humans (and presumably other animals as well) have two separable components of decision-making, one which is impulsive and depends on reacting to immediate, concrete rewards, and another which is more rational and capable of waiting for larger, more abstract rewards (54–57). Importantly, the impulsive (often called “reactive”) system is not necessarily always chasing positive rewards; it can also avoid negative consequences (58), nevertheless, the key difference in the two dual-process hypothesis is that the impulsive system attends to immediate consequences while the other (cognitive, often called “reflective”) system takes into account farther future consequences (59–62). In many of these discussions, the impulsive system is identified as more “emotional” and more related to an animal’s history, while the rational system is identified with more cognitive processing. In many of these theories, the rational system is assumed to be a self-control system, which inhibits the activity of the impulsive system (63–65), often referred to as a form of “self-control” (66, 67). This theory has a very long history (68–70) and there are good summaries of the modern perspectives on this dichotomy (40, 59, 63, 65, 67, 71). Anatomically, the impulsive system is associated with the nucleus accumbens and amygdala, while the rational system is associated with the prefrontal cortex (54, 56, 57, 59, 72, 73).

Recent computational work examining how agents process information to make a decision (such as taking a drug or not) suggests that multiple action-selection systems compete and interact to produce that decision. Current theories suggest that decisions arise from as many as four separable systems, each depending on different information-processing computations (37, 42, 43, 74–77). Each system uses past experience differently and processes information about the world differently, and thus each has advantages and disadvantages in different situations. An agent that correctly identifies the best action-selection system to use in a given situation will outperform a different agent that does not. Because different systems drive behavior at different times, valuation is not necessarily self-consistent.

Following these recent taxonomies (43), we identify four decision-making systems each of which selects actions through a different computation: (1) reflexes, in which evolutionarily useful stimulus–response pairs are hard-wired within a neural system (78, 79), (2) Pavlovian actions, in which an animal learns when to release a species-specific behavior (80–82), (3) procedural actions, in which arbitrary action chains are stored and released on cue (83, 84), and (4) deliberation, which entails a slow, goal-oriented search and evaluate process (42, 85–87). Each of these systems is instantiated in a different anatomical network – reflexes in spinal cord and brainstem (88), Pavlovian actions with amygdala and the periaqueductal gray (89, 90), procedural with motor cortex, cerebellum, and the basal ganglia (91–93), and deliberation with hippocampus and the prefrontal cortex (87, 94–96).

There are many similarities between the dual-process and multiple decision-making systems theories, particularly in the separation between more automatic and more cognitive systems (40, 43, 65). Both theories, for example, suggest that stress and cognitive load will disrupt the more cognitive systems, shifting behavior to more automatic systems. Both theories suggest that the more automatic systems tend to react to more immediate stimuli, while the more cognitive system is capable of incorporating information that is not immediately present.

However, there are important differences between the theories. For example, the information-processing theories do not imply that the more automatic systems are more impulsive, as hypothesized by the classical dual-process distinction. For example, a fire chief with extensive expertise is using a fast, non-deliberative process to make the right choice (83); no one would argue that a fire chief is making an impulsive choice. The more recent models have shown that intuition and developed expertise arises from a different computational process than emotion, suggesting that these are different systems (43). Additionally, the information-processing theory provides for interacting components that can make cognitive systems react differently in the face of concrete stimuli (97, 98).

In addition, the hypothesized causes of addictive behavior is different in the two theories, which has implications for how contingency management should be used and what modifications would do to its success. These subtle differences between these theories make different predictions and change some of the implications of our fundamental hypothesis (that contingency management accesses deliberative processes, see below). We will address the differences between these theories below, but first we address the main implications of our hypothesis that contingency management accesses deliberative processes, which are similar under the two theories.

## 5 Hypothesis: Contingency Management Accesses Deliberative Systems

Our hypothesis is that the provision of a concrete, identified, alternative reward in contingency management both engages deliberative processes and improves the ability of those deliberative processes to attend to non-drug options. In a sense, contingency management transitions the drug-valuation process from a willingness-to-pay condition to a revealed-preference condition. In addition, we propose that the concrete and more immediate rewards provided by contingency management increase the ability of deliberative systems to attend, value, and select the alternative (non-drug) reward. (This may be why the prize-based CM systems are more effective with lower value rewards than comparably more expensive monetary-based voucher systems.)

### 5.1 Pre-clinical experimental support for this hypothesis

Non-human animal self-administration studies have also found that drugs are economic objects and show a non-zero elasticity. As with human studies, increasing the cost (measured in terms of number of lever presses required to receive drug) decreases the number of self-administered drug-taking events (28, 99–101). Similarly, providing an alternative reinforcer reduces the amount of drug self-administration in both rats and monkeys (23–25, 27, 48, 53, 100, 102–106). These studies fall into two categories, which require dramatically different levels of alternative reward to decrease drug use.

Classically, the simplest measure of the cost-dependence of drug self-administration in non-human animals is the *breakpoint* analysis (52, 99). These studies find that much larger costs are required before an animal will cease drug self-administration than before an animal will cease taking non-drug rewards (51, 100). This suggests that it would require very large non-drug rewards to counteract drug self-administration. The first set of studies (24, 25, 27, 104) confirmed this hypothesis, in that they used single-response conditions and found that reductions in drug self-administration were only observed after very large alternative rewards. For example, Woolverton et al. (27) found that the opportunity cost of the drug option needed to be increased 100-fold (for low-drug concentrations) to 1000-fold (for average and high-drug concentrations) in order to significantly reduce self-administration. In these studies, animals could switch between conditions that either provided cocaine on pressing the primary lever or alternative reward on pressing the same primary lever. In other words, the animal could switch between situations that enabled non-deliberative processes. Other studies using similar techniques have found similar proportions (24, 25, 107, 108).

Interestingly, Ahmed [(48), see Ref. (100, 105, 106)] found much smaller alternatives could reduce drug self-administration. In these studies, the animals had two options directly available to them on opposite sides of the chamber – one lever provided cocaine, while the other provided saccharin. Preference was measured by whether the animals selected the saccharin lever or the cocaine lever. These studies also examined single-option breakpoints, in which only one lever was provided and cost was measured as the number of lever presses required before the animal gave up. These studies found that although the breakpoints for cocaine were much higher than the breakpoints for saccharin, animals preferred saccharin when provided with a revealed-preference two-lever choice paradigm. Similarly, LeSage (53) showed that providing a small amount of sucrose for not self-administering nicotine was sufficient to reduce the number of nicotine responses.

These studies support the proposed dichotomy between willingness-to-pay valuations (measured by single-lever breakpoint studies and situation-change studies, theoretically dependent on non-deliberative processes) and revealed-preference valuations (measured as forced choices between two explicit levers). The revealed-preference studies required much smaller rewards to decrease drug self-administration than the willingness-to-pay studies. The difference in size of alternate reward required to change behavior under the two paradigms suggests that the difference lies in fundamental processes underlying decision-making across multiple species (including at least rats, monkeys, and humans).

## 6 Components of Contingency Management That Affect Deliberation

The information processing that underlies deliberative decision-making processes is now beginning to be elucidated (87, 98, 109), particularly, in contrast to other decision-making systems (39, 43, 110). Deliberation requires recognition of a situation, a serial consideration of the potential actions available, and evaluation and comparison of those potential options (42, 87).

The main advantage of deliberation is that because these expected consequences are represented during the decision process, they can be evaluated during that process, in the context of the agent’s current goals (86). This means that the individual options must be found (85, 98, 111, 112) and then the valuation constructed (40, 47, 73, 113). Both the search process and the construction of value will be modulated by processes that computationally affect neural information processing (98, 114). Examples of these include working memory abilities (57, 115), whether the consequence is phrased as a win or a loss (40, 47, 116, 117), attention (113, 118), emotional state (119), surrounding options (120), and even the presence of unrelated numbers, such as in anchoring [where unrelated anchors such as one’s social security number can be used to change one’s expected cost and thus one’s willingness to pay for a reward (40, 47, 117, 118)].

The deliberative process is slow and computationally intensive, likely because of the cumbersome memory-retrieval and imagination-construction system needed to calculate the possible outcomes in order to evaluate them (83, 87, 98, 112). The evaluation achieved through deliberation depends on a number of stimulus factors, including the expected delay to the reward (121), and the concreteness of the reward (97). Deliberation also depends on a number of internal factors, such as one’s perceived needs and desires (86, 119), as well as one’s cognitive and executive-function abilities (98), such as episodic future thinking (95, 96), working memory (115, 122), and ability to hold attention (123, 124).

Valuation derived from deliberation depends on a direct imagination of expected outcomes and a comparison between choices (87, 98, 109). As the preclinical studies reviewed above show (48, 53), when an explicit choice between the drug and non-drug reward options is available, the drug option is less likely to be chosen; therefore, factors that increase the likelihood of engaging deliberative processes or that increase the deliberative valuation of a non-drug option should increase the efficacy of contingency management.

### 6.1 Delay to reward

Rewards that are only available in the future are less valuable than rewards provided immediately (125–127) – something could happen between now and the time one expects to receive the reward (thus diminishing the usefulness of that reward) and immediate rewards can be invested (thus increasing the usefulness of immediate rewards). The diminishing value of future rewards relative to immediate rewards is quantifiably measurable through questionnaires in which subjects make decisions between immediate and delayed amounts of money, drug, or both (121).

Drug users reliably show faster discounting rates than non-addicts (128–132). Recovered addicts, however, show normal discounting rates (128). Although this early study was unable to determine whether this was a selection process in which the addicts with more normal discounting rates responded better to treatment, a more recent study has determined that successful treatment has the effect of normalizing over-fast discounting rates (133).

Many theoreticians have suggested that these preferences for more immediately available rewards can drive drug use because drugs provide very strong immediate rewards (euphoria, relief from dysphoria) while abstinence provides only long-term rewards (health, family, financial) (134, 135). Contingency management may have the effect of bringing the long-term rewards closer by providing more proximal rewards for abstinence (money, vouchers, draws from the prize-bowl).

Given the actual discounting rates reported in realistic subjects (128, 130, 136), $2.50 for the first drug-negative sample would be discounted quickly and seems unlikely to be able to deflect the user away from drugs, especially in the beginning of contingency management treatment. The delay-discounting rates that would be necessary to make these small rewards provided at the end of a week strong enough to affect decisions made days earlier in the week are unreasonably slow (137, 138), particularly for addicts, who have faster discounting rates than non-addicts [for review, see Ref. (28)]. Studies have shown that individuals discount smaller values more quickly than larger values [discounting curves are steeper, Ref. (139)], which would further reduce the discounted effectiveness of the small rewards provided early in treatment.

Furthermore, both human and non-human subjects tend to show hyperbolic discounting functions (121, 140, 141). Any non-exponential (including hyperbolic) discounting function will show *preference reversals* in which one choice is preferred when both choices are far in the future, but the other becomes preferred as the subject approaches the time of that second choice (142). Thus, even if a user decided at the beginning of the week to prefer the contingent reward ($2.50) to taking drugs, when faced with the immediate choice, the user would seem likely to choose the drug-use option.

During treatment in prize-based contingency management, upon submission of a drug-negative sample, individuals immediately earn a chance to win a tangible prize. In addition, individuals have a chance (albeit low in probability) to win a high-value prize for every draw they earn. This means that even though the average overall value of reinforcers earned by subjects tends to be lower in prize-based contingency management compared to voucher-based contingency management, the availability of a more immediate reward and the chance to win a high-value prize may cause individuals to discount less. These differences in discounting rates between the two versions of contingency management may help to explain similar treatment efficacy even with differing value of total potential reward.

### 6.2 Concreteness

The long-term rewards of abstinence tend to be more abstract than the short-term reinforcement provided by drug use (135). Several authors have suggested that the major difference between immediate rewards and delayed rewards is the concreteness of immediate rewards and the abstractness of delayed rewards (98, 143, 144).

Trope and Liberman (143) suggest that high-temporal distance creates difficult-to-conceptualize (high-level, more abstract) construals that are more difficult to reason about, while low-temporal distance creates easier-to-conceptualize (low-level, more concrete) construals. They hypothesize that more concrete options are considered to be more valuable than more abstract options. For an addict, abstinence is a high-level construal placed in the hard-to-imagine far future and is more abstract and less valuable than a concrete reinforcer, such as the option to use drugs in the present or near future, which is a low-level construal.

Current decision-making theories suggest that evaluating future outcomes depends on constructing episodically-imagined futures (87, 109, 113, 145). Kurth-Nelson and Redish (98) suggested that discounting rates may depend on how difficult it is for this construction process to find those potential future possibilities. Supporting this hypothesis is evidence that fronto-parietal areas are more active when people select the delayed option (56, 57), that subjects with better working memory and higher IQs tend to discount more slowly (115), and that training working memory can slow discounting rates (122, 133). Rewards placed in concrete episodic futures (35€ on vacation in Paris next month) are discounted more slowly than abstract future rewards (35€ next month) (146). Kurth-Nelson and Redish (98) suggest that the decreased discounting of concrete options is due to concrete futures being easier to find and construct in the deliberative search process.

Taken together, these theories imply that more concrete rewards have higher subjective value compared to abstract rewards. What does this mean for addiction? Typically, an addict has a choice between using a drug and not using a drug. The option of using the drug has immediate and concrete rewarding effects. Drug’s rewarding effects include subjective pleasurable effects and relief from withdrawal, and both of these effects are expected and concrete. The option of *not* using has immediate negative effects (147), but the primary distal rewarding effects are very abstract (135).

Contingency management changes this scenario by providing the addict with a concrete reward (money, a voucher, a specific prize) contingent upon abstinence, which is more proximal than rewards for abstinence alone. This allows the addict to achieve the goal of reducing drug consumption and increasing abstinence by focusing, not on the abstract abstinence, but rather on the concrete alternative.

This theory suggests that one effect of contingency management is to make both options immediate and concrete. The combination of the discounting/proximity and the concreteness theories suggest that contingency management creates a situation where the alternate reward (i.e., abstinence over drug use) is both more concrete and closer in temporal distance; thus, making it more equal to the drug-use option.

The importance of concreteness is highlighted by comparing voucher- and prize-based treatments. Although subjects were encouraged to imagine concrete items that the voucher could be used for (5), in prize-based studies, the prizes are physically present in a show-cabinet right there with the prize-bowl (9). Vouchers were also useable for a variety of rewards, while winning a given prize meant that that was the concrete prize you got.

Both voucher- and prize-based have been found to be similarly effective, even though the value of possible earned rewards is much lower in the prize-based studies (5, 9, 11, 12, 148). In both versions, high-value rewards have been found to be more successful than low-value rewards; however, the size of rewards offered in these conditions differs considerably. Even though the total value of possible rewards received in the high-value prize-based method was lower than the low-value voucher-based method, the high-value prize-based method was still effective for significantly reducing drug consumption, while the low-value voucher-based method was not. This not only exemplifies the importance of value but also how the concreteness of the reward affects perceived value. The presence of more concrete alternative rewards (specific prizes) appears to have more of an effect than less concrete alternative rewards (voucher exchanged for money, in turn, used for unspecified merchandise).

## 7 Conclusion and Further Discussion

In summary, we propose that contingency management’s success occurs because it provides an alternate reinforcer that forces the subject into a deliberative mode, which allows different valuation processes than non-deliberative modes. It also provides both a decreased time-to-reward and increased concreteness for the alternate reward, which should increase the valuation of the alternate reward relative to the valuation of the drug and move the agent from a willingness-to-pay valuation mode to a choice between/revealed-preference valuation mode.

### 7.1 Relationship to classical dual-process theories

Many theoreticians have suggested that addiction arises from a mismatch between the balance of two systems (typically called a “hot” or impulsive system and a “cold,” rational system) (64, 149, 150). While it is possible to place our hypotheses for contingency management within that two-system framework, we believe that the evidence suggests that addiction is more complicated than the simple out-of-balance theory proposes. Instead, we work from the theory that continued drug use can arise from computation errors in a number of places within the decision-system, of which a mismatch in balance between systems is only one potential failure mode (39, 43).

It is important to differentiate the *vulnerabilities* theory of addiction that arises from the *multiple action-selection-system* theory from the *out-of-balance* theory of addiction that arises from the *dual-process* theory. (See Table 2 for a list of these decision-concepts used in this paper.) Our proposal that contingency management drives subjects toward deliberative processes could follow from either of these two addiction/decision-making theories, but the implications are different, depending on which theory pertains.

**Table 2:** **Economic theoretical constructs used in this article**.

**• Dual-process theory**: the idea that there are two decision-making systems, an impulsive system and a rational system
**• Out-of-balance theory**: the idea that addiction arises from an imbalance between the impulsive and rational systems
**• Multiple action-selection system theory**: the idea that there are multiple ways to select actions from information about the world (cues), history (memory), and goals (needs/desires). Each of these systems is optimal in different situations
**• Vulnerabilities theory**: the idea that addiction arises out of processing failures in one or more of the action-selection systems

The *out-of-balance* hypothesis of addiction is that addicts have a problem with the balance between the two systems in the dual-process theory (54, 55, 66, 67, 149). These systems can be driven out of balance either from hyperactivity in the impulsive system or hypoactivity in the rational system (55, 56, 151, 152). In either case, improving the strength of the rational system [for example, by providing working memory training (122) or by increasing activity in the prefrontal cortex (153)] should decrease drug use because it should shift the balance toward the more rational system. Our proposal that contingency management drives decision-making toward deliberation implies that if the dual-process and out-of-balance theories are correct, then what contingency management is doing is shifting the balance between these two systems. Evidence supporting this concept was recently published by Wesley et al. (57), who found that in an explicit cocaine-money choice, choosing money later over cocaine now produced additional activity in the dorsolateral prefrontal cortex.

The vulnerabilities hypothesis of addiction is that there are many potential “failure modes” within these systems, any of which can lead to addictive behaviors (39, 43, 77, 154). The concept that there are many vulnerabilities implies that addiction can arise from multiple causes. Our proposal that contingency management drives decision-making toward deliberation implies that if the multiple-action-selection systems and vulnerabilities theories are correct, then what contingency management is doing is twofold: (1) it is shifting the decision-making system into deliberation because it is providing two choices, and (2) it is improving the deliberation system algorithm, by making the goals more concrete and more immediate.

There are similarities and differences between these theories. Both theories include separate action-selection systems, only one of which includes an explicit planning component.

- The concrete nature of the alternative reward in contingency management is going to access that planning component, driving behavior toward it.
- Under neither hypothesis is the alternative reward fast enough to access the non-planning systems.
- In both theories, the planning-capable system depends on cognitive resources and prefrontal cortex.

However, the vulnerabilities theory further proposes that there are failure modes within the deliberative system as well, and thus suggests that only a subset of patients will be helped by contingency management, and that different aspects of contingency management will help different patients.

- For patients who have vulnerabilities in the Pavlovian or procedural systems who may express a desire to quit in the absence of drug-related cues, but find themselves unable to when faced with drug-related cues, contingency management can provide a second option to attend to, even when faced with drug-related cues, which can enable the deliberative system to retain control. This likely relates to the difference in valuation between single-option choices (go/no-go, willingness to pay) and dual-option choices (select between).
- For patients who have vulnerabilities in the evaluation step of deliberative systems, the concrete nature of the alternative reward in contingency management can make that reward easier to locate in the search-through-the-future process. This likely relates to the dependence of the search process on episodic future thinking.
- For patients for whom the drugs are simply an alternative reward option or for patients who have limited access to alternative rewards (155), then the opportunity cost provided by contingency management could be enough to make them reject the drug option.
- Because the vulnerabilities theory proposes that some patients will have vulnerabilities within the deliberative decision-making system [such as incorrect hypotheses about consequences of their actions (156, 157)], these patients will not be helped by contingency management, at least until they address those deliberative deficiencies.

### 7.2 Predictions and implications

#### 7.2.1 Identify Patients Capable of Deliberating

The idea that contingency management primarily accesses deliberative systems implies that it will be most successful in patients with viable deliberative systems. This suggests that identifying patients with intact deliberative systems would help identify patients most likely to be helped by contingency management programs. There are a number of cognitive tasks known to access deliberative systems (94, 146, 158–161). Whether these tasks are changed in addicts, however, remains unknown. The vulnerabilities theory predicts that some addicts will continue to show deliberative abilities in these tasks, and that those addicts will be best served by contingency management.

This hypothesis further suggests that patients with deficient deliberative systems would be helped by first training those systems. Working memory training, for example, decreases discounting rates as much as drug treatment (133).

#### 7.2.2 Prediction: Contingency Management will Depend on Prefrontal Integrity

The two hypotheses that contingency management depends on deliberative processes and that deliberative processes depend on prefrontal integrity predict that contingency management will be most successful in patients with strongly active prefrontal systems. Evidence that prefrontal cortical interactions with hippocampus and other neural systems are a necessary component for deliberative decision-making processes is well-established (71, 94, 96, 145, 162, 163). For example, functional connectivity between prefrontal cortex and nucleus accumbens predicts success in drug-dependence treatment and an avoidance of relapse (152). In rats, optogenetic stimulation of prelimbic (prefrontal) cortices decreases compulsive drug seeking, while optogentic inhibition of prelimbic (prefrontal) cortices increased it (153). Similarly, in humans, repetitive transcranial magnetic stimulation (rTMS) over the dorsolateral prefrontal cortex reduced reported craving in nicotine addicts (164).

It also suggests that patients with improved cognitive abilities (115) and with prefrontal cortices more likely to play active roles in decision-making (56, 57, 152) will be more capable of using contingency management. These hypotheses imply that further improvements in cognitive resources [such as with working memory training (122, 133)] or increasing prefrontal activity (153) will make patients be more capable of using contingency management.

#### 7.2.3 Combine Contingency Management with Working Memory Training and Cognitive Reassessment Therapy

Contingency management is often provided with synergistic treatment of pharmacological and sociological treatments (counseling, 12-step group work, methadone or nicotine-replacement treatment, etc.) (1). While these additional treatments provide potential rectification of decision-making vulnerabilities and failure modes, we suggest that they do not directly address the reasons for the success of contingency management. Under the hypothesis that contingency management depends on deliberative processes, improvements in those deliberative processes should provide additional improvements in the success of contingency management.

Deliberative decision-making entails the creation and imagination of hypothetical episodic futures and evaluation of those futures (43, 85, 109, 111, 145). As such, it requires a search process and memory to compare those evaluations (87, 98, 163). Changes in the recognition of the underlying paths through those futures affect the decisions made (138, 140, 165). For example, the famous dictum that “there is no such thing as one drink for an alcoholic” implies that decisions are not between drinking one drink and not, but between drinking many drinks and not. This process leads to *bundling*, in which future decisions are bundled together, which changes the underlying valuation of those future decisions (140, 165).

Changes in the ability to create, imagine, test, and remember those futures will also likely increase the ability to engage that deliberative system. It is possible to improve executive function and working memory through training (122, 166). These procedures decrease impulsivity as measured by discounting experiments. Given the data that cognitive load decreases engagement of the deliberative system (67, 124, 160), merely recognizing that patients are particularly vulnerable under stress and situations of increased cognitive load (165, 167), could suggest proactive procedures (such as increased rewards or increased reminders) during times of stress and cognitive load.

#### 7.2.4 Increasing Value of the Alternate Option

From the very first introductions of contingency management, it has been clear that providing an increased value of the alternate rewards increases the success rate (1, 11, 148). This is a straightforward prediction of the alternate reinforcement theory. However, as expected from the discussion of the pre-clinical data (above), dramatic changes would require very large alternate rewards. For example, increasing the payout from $0.50 on the first negative drug urine sample to $7.00 produces a significant effect (168). Given the political difficulty of paying for drug treatment programs, finding ways to increase the success of contingency management without dramatically increasing costs would be particularly useful. Prize-based contingency management is one example of reducing costs without decreasing efficacy (1, 11).

#### 7.2.5 Concrete Options are Discounted Less than Abstract Options – Provide Reminders of the Concrete Alternate Reward

If one could increase the proximity of the rewards at the moment of decision, one could further increase the value of the alternative option. Thus, one potential improvement would be to provide a concrete reminder of the alternate reward (such as what the current voucher value is) on an easily accessible place (such as a smartphone app) that could be accessed at the actual moment of decision.

Although concrete options are more valuable than abstract options, symbolic reminders of concrete options might also increase the value of alternate options. For example, simply stating a delayed reward will be delivered during an episodic event decreases discounting and increases value relative to equivalent, but less concrete rewards (146). Similarly, pictures of food rewards are more valuable than text descriptions of those rewards (169). Thus, visual symbols can improve both concreteness and deliberation. This suggests that providing the picture of the specific concrete option being worked toward is likely to further improve the reminder. Similarly, providing direct information about the values of the alternative options (such as days clean, days remaining to reward, points that would be lost due to relapsing) would make it easier for the patient to evaluate the alternative outcome, which should make it easier for the patient to attend to (and select) the alternative outcome. This could also be accomplished through a smartphone app that shows the picture of the reward being worked toward and information about the voucher points needed to achieve that goal.

#### 7.2.6 Preventing Relapse after Contingency Management Treatment

As with any treatment, many patients relapse after treatment. The vulnerabilities theory suggests that addiction is caused by a multitude of potential failure modes (39, 43). Although contingency management is a support mechanism that can aid in a person’s recovery, other failure modes may still remain even after completion of the contingency management series. However, contingency management can be combined with other treatments (1, 5, 9). Studies have shown that the cognitive and discounting impairments that arise during drug and alcohol use improve with continued abstinence (133, 170–174). Thus, contingency management can create a span of time for an individual to repair these failure modes, while also learning important skills to increase the chance to remain abstinent in the future.

One potential solution would be to teach users to create their own contingency management process, providing their own deliberative alternatives. Changes in expectations and representations of the outcomes of potential options can change decision-making choices, even without changes in the underlying action-selection processes (135, 138, 140).

## Author Contributions

The manuscript was co-written by both authors.

## Conflict of Interest Statement

The authors declare that the research was conducted in the absence of any commercial or financial relationships that could be construed as a potential conflict of interest.

## Footnotes

^1^In the Higgins et al. (5) study, subjects got a voucher worth $2.50 for the first clean sample. Taking this voucher as covering staying abstinent for more than 1 day would only decrease the predicted impact of the voucher. Since our analysis will show that voucher size is inadequate to drive changes in the demand curve, any increased required abstinence will not change our conclusions.
